# Orthopedia Homeobox (OTP) in Pulmonary Neuroendocrine Tumors: The Diagnostic Value and Possible Molecular Interactions

**DOI:** 10.3390/cancers11101508

**Published:** 2019-10-08

**Authors:** Laura Moonen, Jules Derks, Anne-Marie Dingemans, Ernst-Jan Speel

**Affiliations:** 1 Department of Pathology, GROW School for Oncology and Developmental Biology, Maastricht University Medical Centre, 6229HX Maastricht, The Netherlands; ernstjan.speel@mumc.nl; 2 Department of Pulmonary Diseases, GROW School for Oncology and Developmental Biology, Maastricht University Medical Centre, 6229HX Maastricht, The Netherlands; j.derks@maastrichtuniversity.nl (J.D.); a.dingemans@mumc.nl (A.-M.D.)

**Keywords:** orthopedia homeobox, pulmonary carcinoid, neuroendocrine tumor, prognosis, classification, review

## Abstract

Generally, patients with stage I-IIIa (TNM) pulmonary carcinoid disease have a favourable prognosis after curative resection. Yet, distant recurrence of disease after curative surgery occurs in approximately 1–6% of patients with typical carcinoid and 14–29% in patients with atypical carcinoid disease, respectively. Known predictors of distant recurrence of disease are atypical carcinoid, lymphatic involvement, and incomplete resection status. However, none of them can be reliably used, alone or in combination, to exclude patients from long-term follow-up (advised 15 years). By genomic profiling, Orthopedia homeobox (*OTP*) has been identified as a promising prognostic marker for pulmonary carcinoid with a favourable prognosis and low risk of distant disease recurrence. Moreover, OTP is a highly specific marker for carcinoids of pulmonary origin and recent genome wide analysis has identified *OTP* as a crucial predictor of aggressive tumor behaviour. OTP in combination with CD44, a stem cell marker and cell-surface protein, enables the identification of patients with surgical resected carcinoid disease that could potentially be excluded from long-term follow-up. In future clinical practice OTP may enable clinicians to reduce the diagnostic burden and related distress and reduce costs of long-term radiological assessments in patients with a pulmonary carcinoid. This review addresses the current clinical value of OTP and the possible molecular mechanisms regulating OTP expression and function in pulmonary carcinoids.

## 1. Introduction

Pulmonary carcinoids (PC) are rare, well-differentiated neuroendocrine tumors accounting for 1–2 per cent of all lung cancers [1]. Nevertheless, its occurrence has increased significantly over the past decades (approximately 6 per cent per year over the past 30 years in both men and women) [2,3]. In contrast to high-grade neuroendocrine lung carcinomas, such as large cell neuroendocrine carcinoma (LCNEC) and small cell lung cancer (SCLC), carcinoids are characterized by a lower metastatic rate and a relatively favourable prognosis. According to the World Health Organization (WHO), PC are recognized morphologically by a neuroendocrine growth pattern and can be subdivided into typical carcinoids (TC) and atypical carcinoids (AC) based on the mitotic rate (less than 2/mm^2^ for TC and between 2 to 10 for AC) and the presence of necrosis in ACs [4]. Although TCs and ACs are considered low- or intermediate-grade tumors, they may spread to regional lymph nodes and distant organs. TCs are characterized by a relatively favourable prognosis (5–20% metastasize), whilst ACs are more often characterized by a malignant behaviour and a lower 5- and 10-year survival rate (30–40% metastasize) [3]. Although curative treatment by means of surgical resection is possible for most carcinoids, distant disease recurrence may still occur even up to 20 years after curative treatment [1,5]. More precisely, distant recurrence of disease after curative surgery ranges from 1–6% for typical carcinoids and 14–29% for atypical carcinoids [6,7,8,9,10]. In current practice, recurrence in these patients is not predictable due to a lack of clinical-pathological features that enable accurate prediction of disease recurrence. As a consequence, all patients with PC require radiological assessment and follow-up for an extensive period of time (15–20 years) [1]. 

The requirement of surgical tissue for assessment of all diagnostic morphological criteria for PCs limits accurate diagnosis on small biopsies and cytology specimen. Besides histological classification, recent literature described the Ki-67 proliferation index as a valuable marker to distinguish carcinoid tumors (<20%) from high grade LCNEC and SCLC (≥20%) on biopsy specimens [3,4,11,12]. However, the utility of this marker to differentiate TCs from ACs or to predict prognosis within individual carcinoid tumors is limited [13]. These diagnostic limitations indicate the need for alternative molecular markers to subdivide carcinoids into clinical relevant categories [4]. 

By expression profiling, Orthopedia Homeobox (*OTP*) has recently been identified as a reliable molecular marker to predict the prognosis of PC patients [14]. Although *OTP* has frequently been described as a key player in the development of the hypothalamic neuroendocrine system of vertebrates, its function in lung carcinoids remains to be elucidated. Here, we comprehensively review current literature on OTP function, OTP expression in lung neuroendocrine neoplasms, and possible molecular pathways through which it might operate in both normal and pulmonary neuroendocrine tumor tissue. 

## 2. OTP in Relation to Prognosis in PC

Though the role of OTP in pulmonary carcinoids is poorly understood, it has been described as a strong prognostic marker for pulmonary carcinoids [14,15]. In 2013, Swarts et al., reported the molecular characterization of carcinoids in patients with prolonged and poor survival rates (*n* = 10 discovery, *n* = 54 validation) [12,14]. Results revealed a set of downregulated genes in the unfavourable group, one of them showed a remarkably strong downregulation namely *OTP* (median fold change of 845) [16]. Multivariate analysis, comparing *OTP* with clinical parameters, showed that loss of or decreased expression of *OTP* was independently associated with unfavourable survival and increased risk of metastases. These findings were validated at the protein level by immunohistochemistry using the rabbit-anti-OTP polyclonal antibody (clone HPA039365, Atlas Antibodies, Stockholm, Sweden) [14]. The prognostic value of OTP has since been validated in larger series (*n* = 288), confirming that loss of expression is associated with poor prognosis (Table 1 and Table 2) [15,17]. 

**Figure 1:**
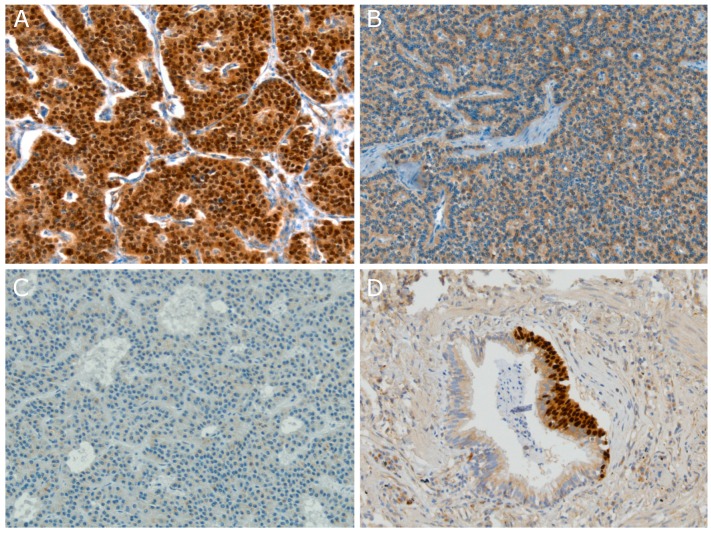
Orthopedia homeobox (OTP) immunohistochemistry of pulmonary carcinoids and neuroendocrine cell hyperplasia (NECH). (**A**) Representative image of nuclear OTP (nOTP) and cytoplasmic OTP (cOTP) staining in a typical carcinoid; (**B**) Representative image of a carcinoid tumor harbouring only cytoplasmic immunoreactivity for OTP; (**C**) Representative image of a carcinoid tumor with no OTP immunoreactivity; (**D**) Representative image of both nOTP and cOTP staining in NECH (magnification 200×) [14,16].

Three different OTP expression patterns can be observed and are strongly related to patient outcome namely, a strong nuclear staining (nOTP) with or without cytoplasmic reactivity, an exclusively cytoplasmic staining (cOTP), and a negative staining pattern (Figure 1A–C) [14]. Patients with nOTP expression have a favourable disease outcome, patients with cOTP reactivity have intermediate survival, and patients with absence of OTP expression rendered the worst disease outcome [14]. In addition, Swarts et al., showed a strong correlation between absence of nOTP and the occurrence of distant metastasis (*p* = 0.00014) [14]. Most interestingly, we observed that OTP in combination with CD44, a cell-surface glycoprotein involved in cell-cell interactions, allowed for even better separation of tumors into prognostic relevant categories. These results have been independently confirmed by Papaxoinis et al., evaluating 86 cases [15]. Results showed that CD44 and nOTP staining were an independent predictor for relapse-free survival (RFS) in patients with radically operated pulmonary carcinoids (Hazard Ratio (HR) 0.192, 95% Confidence Interval (CI) 0.064–0.574; *p* = 0.03) [15]. No statistically significant differences in relapse-free survival were observed for patients with tumors containing decreased expression of one or both proteins (*p* = 0.861). For this reason, Papaxoinis et al., proposed that a combination of CD44 and nOTP staining may be a prognostic marker to predict prognosis and development of recurrence of disease. To date, no evidence for a molecular interaction between OTP and CD44 has been reported.

Current studies indicate that protein expression of OTP/CD44, mainly nuclear staining, can be used to stratify PCs into prognostic relevant subgroups independent of the morphological established diagnosis [14,15]. In addition, due to the morphological similarities, the existing histology-based grading is problematic and subject to considerable interobserver variation. Swarts et al., examined the interobserver variation among five experienced pulmonary pathologists who reviewed 123 originally diagnosed pulmonary carcinoid cases [22]. In total 114 of the 123 cases were unanimously classified as pulmonary carcinoids. Fifty-five percent (63/114) were unanimously classified, 25% (29/114) reached consensus classification, and no consensus was reached for 19% (22/114), which comprised predominately ACs [22]. Although, consensus reclassification may improve prediction of survival of pulmonary NETs, it did not improve the prediction of prognosis of the disagreement cases (from *p* = 0.11 to *p* = 0.14) [22]. Nevertheless, when disagreement cases were allocated on the basis of nOTP immunostaining patient prognosis prediction improved significantly (from *p* = 0.11 to *p* = 0.0024). Thus, molecular markers may be used to classify carcinoids into prognostic relevant categories. Future studies should evaluate the diagnostic sensitivity and specificity of OTP as a marker to predict metastatic disease after curative surgery and if this marker is applicable for diagnostic and prognostic stratification on biopsy specimens.

## 3. OTP Is Specifically Expressed Within Pulmonary Carcinoids

Whilst OTP has been described as a prognostic marker for PCs, little is known about the expression pattern of this transcription factor in normal organs/tissues and other tumors. OTP expression in other neuroendocrine tumors and/or normal tissue and organs has been investigated in six studies [14,17,18,20,21,23]. Swarts et al., previously analysed the expression of *OTP* by qRT-PCR on frozen material of carcinoids, LCNEC, SCLC, normal tissues, and neuroendocrine cell lines (Bon-1, CM, NCI-H69, NCI-H295, NCI-H460, NCI-H720, NCI-H727, QGP and SW13). Carcinoid tumors showed a positive *OTP* messenger-RNA (mRNA) expression whereas normal tissues, high-grade neuroendocrine carcinomas, and the evaluated NE cell lines did not express *OTP* mRNA [14]. In addition, Nonaka et al., investigated immunohistochemical OTP expression in a variety of tumors, with special interest in pulmonary and non-pulmonary neuroendocrine tumors, neuroendocrine carcinomas, and normal tissues and organs [17]. Nuclear OTP expression was observed in 80% (130/162) of all pulmonary carcinoid tumors. Four out of 34 small cell carcinomas showed focal expression of OTP whereas all other tumors were completely negative. In line with the findings of Swarts et al., OTP was neither expressed in normal tissues nor in other organs examined [17]. Neuroendocrine cells of the normal bronchus and bronchiole, identified with synaptophysin, were negative for OTP as well. Similar results were observed by Hanley et al., who evaluated immunohistochemical OTP expression in fine-needle aspiration (FNA) samples derived from extrapulmonary and pulmonary sites (Table 1). OTP was positive in 17% (10/59) of the cases, and all positive samples were NETs from either the lung or a metastasis from a primary lung tumor (Table 2) [18]. None of the NETs derived from extrapulmonary sites showed any positivity for OTP. Among the 15 pulmonary carcinoids, 100% (9/9) of the TCs were positive compared to only 17% (1/6) of the ACs, indicating that OTP is preferably expressed within TCs (Table 2) [18]. Nevertheless, Viswanathan et al., evaluated OTP expression in both pulmonary non-neuroendocrine and neuroendocrine tumors (Table 1) [21]. According to the results, neither non-neuroendocrine tumors nor high grade neuroendocrine carcinomas stained positive for OTP. However, 82% (9/11) of TCs and 83% (10/12) of ACs showed positivity for OTP confirming the specificity of OTP for PCs (Table 2). Yet, in contrast to other studies, both TCs and ACs showed equal positivity towards OTP on fine needle aspiration (FNA) specimen [17,18,21]. However, it should be noted that they did observe a significant difference in the degree of OTP staining in surgical resection material between TC and AC. 73% of TCs showed >40% OTP staining, whereas only 30% of ACs displayed >40% OTP staining, indicating that the staining used in this study was suboptimal for small biopsies. 

Another recent study of Yoxtheimer et al., examined OTP IHC expression on 50 FNA specimens, including 30 primary pulmonary NENs (eight TCs, six ACs, five LCNEC, and 11 SCLC) and 20 primary pancreatic NETs (Table 1) [20]. Results showed that 50% (4/8) of the pulmonary TCs expressed OTP, while merely 17% (1/6) of ACs and 20% (1/5) of LCNEC expressed OTP (Table 2). Moreover, neither SCLC nor any pancreatic NET expressed OTP [20]. Taken together these studies define OTP as a highly specific marker for pulmonary carcinoid disease. Since OTP turns out to be a highly specific marker, an increasing number of studies are starting to evaluate the diagnostic utility of OTP in tumors with NE differentiation. Recently, Roy et al., assessed OTP expression in tissue microarrays of 32 FFPE malignant tumors with neuroendocrine differentiation from the gynaecologic organs (*n* = 16), breast (*n* = 8), and prostate gland (*n* = 6) [23]. Nuclear expression of OTP was interpretable in 26 cases and detected in only 15% (4/26) including two prostate adenocarcinoma and two NE carcinomas of the ovary. OTP expression was absent in the remainder of the gynaecologic malignancies and NE mammary carcinomas. These data imply that though OTP is a very specific marker for NE lung carcinoids, it is not a sensitive broad-spectrum NE marker. 

## 4. OTP in Pulmonary Neuroendocrine Cell Hyperplasia (NECH)

Diffuse idiopathic pulmonary neuroendocrine cell hyperplasia (DIPNECH) was first described by Aguayo et al., as an unusual clinical entity that may cause airway fibrosis [24]. DIPNECH is confined to the respiratory epithelium layer without penetration of the basement membrane and is not related to any known predisposing condition [25]. Nowadays, it is known that pulmonary neuroendocrine cell hyperplasia (PNECH) can emerge, not only as a reaction to inflammation, but also in the context of carcinoid or adenocarcinoma development (Figure 1D) [26]. In the latest WHO classification of lung tumors, DIPNECH was described as a generalized proliferation of pulmonary neuroendocrine cells that may be restricted to the mucosa of airways, may invade locally to form tumorlets, or might develop into carcinoid tumors [4]. Interestingly, we found that OTP is highly expressed in these speculated lung carcinoid precursors [16]. In a cohort study containing seven DIPNECH cases, Nonaka et al., showed that OTP was not expressed in normal tissues and organs whilst OTP was convincingly expressed in all DIPNECH lung resections (Table 2). Afterwards, the same research group added an additional nine DIPNECH cases to their former research cohort which showed an ubiquitous OTP staining as well (Table 2) [19]. A recent review illustrated the progression of disease in carcinoids and proposed that carcinoid tumors may arise from neuroendocrine cells and related neuroendocrine cell hyperplasia [27]. In addition, it was suggested that the genes OTP/CD44 might play an important role in carcinoid development.

## 5. OTP Structure

**Figure 2:**
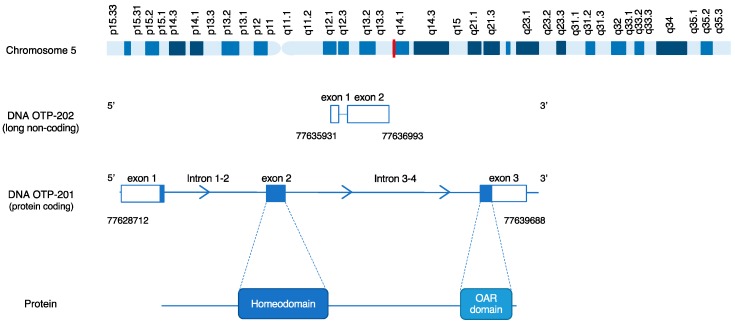
Schematic representation of the *OTP* gene. The chromosome row represents the genomic location of *OTP* (Red, 5q14.1), the DNA rows represent the gene composition with the genomic coordinates of both the protein coding transcript and the processed transcript, and the protein row represents the translated protein domains within the gene.

*OTP* is a gene which encodes a member of the homeodomain (HD) family. A homeodomain is a 180-nucleotide DNA sequence that encodes a helix turn helix DNA binding domain, which was discovered in Drosophila flies during the mid-1980s by McGinnes et al. [28]. Genes possessing a HD are transcriptional regulators which play key roles in the specification of cell fates. In an effort to identify human HD genes, Lin et al., successfully cloned the human homologue of the murine gene Orthopedia (*Otp*), which demonstrated 99% homology to mouse *Otp* [29]. The human gene is located at chromosome 5q14.1 containing three exons and two introns, two splice variants and a high GC-content (Figure 2). *OTP* encodes a protein composed of 325 amino acids and contains two protein domains namely a homeobox domain and an OAR-domain, the function of the latter is unknown (Figure 2). 

## 6. OTP Function

### 6.1. OTP in the Hypothalamus

**Figure 3:**
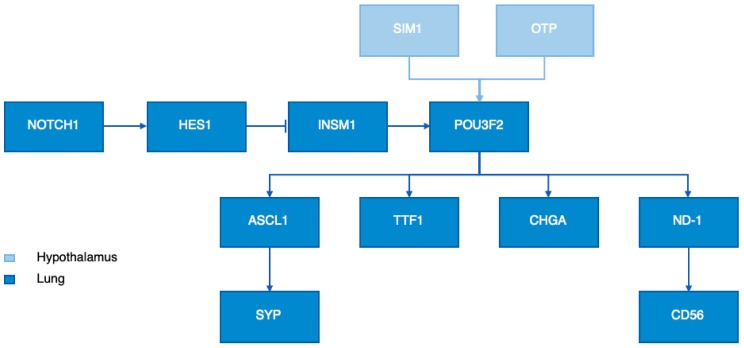
Proposed schematic overview of the molecular network through which OTP might act along with other neuroendocrine (NE) related factors in the hypothalamus and the lungs.

Although the specific function of OTP remains largely unknown, OTP has been frequently reported to be a key player in the development of the hypothalamic neuroendocrine system of vertebrates such as zebrafish, mice, and humans [30,31,32]. The hypothalamic neuroendocrine system is a crucial region in the brain which regulates homeostasis by mediating endocrine, autonomic, and behavioural functions. It comprises several nuclei containing distinct neuronal populations producing neuropeptides and neurotransmitters which regulate fundamental body functions [33]. In mice, Otp expression is well conserved in hypothalamic domains and involved in the differentiation of several neurohormone secreting nuclei including the anterior periventricular, paraventricular, supraoptic, arcuate nuclei [31,34,35]. Neurogenesis of the endocrine hypothalamus is characterized by a series of crucial developmental milestones such as the initial commitment to the neuronal fate, neuroblast proliferation, migration of postmitotic neurons to the neuroendocrine nuclei, and terminal differentiation including neuropeptide expression and axonal outgrowth [32]. Analysis of *Otp* knockout (*Otp-/-*) mice revealed that Otp is able to affect all developmental milestones except for the initial commitment to the neuronal fate, indicating an essential role for Otp in proper murine neuroendocrine hypothalamic development [32,34]. Nevertheless, the development of neuroendocrine cell lineages in the hypothalamus requires, besides OTP, additional transcription factors. Acampora et al., showed that OTP is coexpressed with single-minded-homology 1 (SIM1) in the same cells at the same time [32]. In addition, they showed that Otp acts upstream of Brn-2, a developmental neural cell-specific POU domain transcription factor (POU3F2). By using single-minded-homology 1 (*Sim1*) mutant mice, Acampora et al., showed that both Otp and Sim1 are required for POU3F2 expression (Figure 3). To summarize, OTP is a highly conserved transcription factor which regulates the fate, migration, and terminal differentiation of hypothalamic neurons.

### 6.2. OTP in Lung Neuroendocrine Tumors

A recent study of Nonaka et al., proposed a link between OTP and thyroid transcription factor 1 (TTF1) [17]. The expression of TTF1, a member of the homeodomain transcription factor family, is one of the most essential IHC stains in the diagnostic histopathology of lung and thyroid tumors [36]. Immunohistochemical analysis on 162 pulmonary carcinoids revealed that all TTF1 positive tumors were also positive for OTP [17]. On the other hand, none of the OTP negative tumors stained positive for TTF1. Additionally, neither OTP nor TTF1 was expressed in normal NE cells [15,19]. Taken together, we propose that OTP might be upstream of TTF1 in the transcription factor hierarchy. Nevertheless, Papaxoinis et al., and Hanley et al., described cases positive for TTF1 whilst negative for OTP suggesting the presence of intermediate factors in PCs [17,19]. One of these intermediate factors might be the downstream target of OTP, POU Class 3 Homeobox 2 (POU3F2), since a recent study proposed a crucial role for POU3F2 in the expression of lineage-specific transcription factors such as achaete-scute homolog-like 1 (ASCL1) and NeuroD1 (ND1) and NE marker molecules like neural cell adhesion molecule 1 (NCAM1), synaptophysin (SYP), and chromogranin A (CHGA) in SCLC (Figure 3) [37]. In addition, Sakaeda et al., reported that POU3F2 is directly involved in TTF1 expression in SCLC [38]. 

A possible explanation for the OTP+/TTF1− cases might be the involvement of the NOTCH1-HES1 signalling pathway which is reported as an inhibitor of ASCL1, POU3F2, and NE molecules (CHGA, CD56, SYP) [39]. NOTCH receptor 1 (NOTCH1) is known to activate hairy and enhancer of split-1 (HES1), which inactivates insulinoma-associated protein 1 (INSM1), ASCL1, and POU3F2 (Figure 3) [40]. Nevertheless, recent studies described that Notch1 signalling is minimal or even absent in pulmonary TC and AC and gut carcinoids [40,41,42,43,44]. As a result, the transcription factor HES1 will be inactive and INSM1 will not be inhibited leading to the activation of the transcription factors ASCL1 and POU3F2 which promote the expression of NE molecules such as TTF1 (Figure 3). Although the pathway through which OTP acts remains largely unknown, here we speculate a schematic overview of downstream targets of OTP and the possible involvement of the NOTCH1 pathway. 

## 7. *OTP* DNA Analysis

Several studies extensively profiled PCs to obtain more insights into the molecular characteristics of these rare entities [27,45,46,47,48,49]. Recently, Alcala et al., performed multiomics (genome, exome, transcriptome and methylome) integrative analyses on 116 PCs [50]. Carcinoids were classified into different clusters based on multiomics cluster analysis (MOFA) of which cluster carcinoid A was enriched for typical carcinoids (75%) whilst cluster carcinoid B was enriched for atypical carcinoids (54%) [50]. Cluster B showed the worst survival and was characterized by the universal downregulation of *OTP* (90% with fragments per kilo million (FPKM) < 1). In addition, they showed that these expression levels of *OTP* were strongly correlated with survival which is in line with previous studies [14,15]. Despite the high expression difference between favourable versus poor survival, the regulatory mechanism of *OTP* remains to be further investigated. 

Nowadays, patterns of somatic mutations caused by different mutational processes in cancer genomes have been identified as the result of advances in genome sequencing and the development of computational tools. Multiple studies have performed mutational analysis on pulmonary carcinoid tumors [45,46,47,48,49]. Though carcinoids are characterized by a low number of nonsynonymous mutations per million base pairs, most frequently mutated genes are implicated in chromatin remodelling. In addition, histone modifiers and members of switch/sucrose non-fermentable (SWI-SNF) complexes are mutated in approximately 40% and 22%, with multiple endocrine neoplasia type 1 (MEN1) most frequently affected [45,47,51]. However, until now no mutations have been identified in *OTP*, suggesting the presence of additional mechanisms leading to substantial expression differences. 

Besides genetic changes in DNA and chromosomes, it has become evident that oncogenomic processes can be profoundly influenced by epigenetic mechanisms. DNA methylation is a major epigenetic factor involved in the regulation of gene expression and refers to the addition of a methyl group to the fifth position of a cytosine [52]. Nowadays, numerous human diseases have been linked to aberrant DNA methylation of which hypermethylation of CpG islands in the promotor region has been most extensively studied in cancer. Currently, various genes with aberrant promotor hypermethylation have been identified in all forms of cancer. While several studies have explored promotor methylation in pulmonary carcinoids, to date, only Alcala et al., investigated the whole methylome of 56 carcinoids (33 TCs and 23 ACs) using 850K arrays [50]. Gene expression and corresponding promotor methylation data were correlated to identify genes which expression could be explained by their methylation pattern. While one of the top correlations was found for *HNF1A* and *HNF4A* homeobox genes, no correlation was found for *OTP* [50]. 

In summary, different *OTP* mRNA and protein expression levels are found to be related to prognosis. However, the exact mechanism of OTP (in) activation has not been identified yet. 

## 8. Conclusions

We provide a comprehensive overview of available literature demonstrating OTP as a promising, highly sensitive, and specific marker for pulmonary carcinoid tumors with a favourable prognosis. Nuclear OTP in combination with CD44 protein expression may be used as a predictive marker to exclude patients having a very low risk for distant recurrence of carcinoid disease from long term follow-up. Nevertheless, additional cohort studies focusing on disease free survival are necessary to implement OTP in routine diagnostics. Besides, the underlying mechanism regulating OTP in neuroendocrine pulmonary (tumor) cells remains to be elucidated. Hence, future studies should be focused on unravelling the interplay between regulation of OTP expression, and its biological role in the downstream aggressive behaviour of these pulmonary neuroendocrine lesions. 

## Figures and Tables

**Table 1:** Overview of the characteristics of studies that analysed OTP expression in pulmonary carcinoids using immunohistochemistry.

	Study Population				Histology						
Study, Year [Ref]	Initial Cohort	Included in Analysis	Age (years)	Gender (*n*)	DIPNECH	TC	AC	HGNECs	WHO Classification (year)	Normal Tissue Included (*n*)	Other (NE) Tissues Included (*n*)
Hanley et al., 2018 [18]	63	59	26–91	M(24), F(39)	0	9	6	1	Yes (2015)	No	Yes (51)
Nonaka et al., 2016 * [17]	159	159	21–83	M(62), F(97)	7	123	21	104	Yes (2015)	Yes (*n*/a)	Yes (758)
Papaxoinis et al., 2018 * [19]	166	166	16–83	M(62), F(104)	16	132	34	0	Yes (2015)	No	No
Papaxoinis et al., 2017 * [15]	108	86	21–83	M(44), F(64)	8	69	17	0	Yes (2015)	No	No
Swarts et al., 2013 [14]	352	348	16–83	M(130), F(159)	0	225	63	59	Yes (2003)	Yes (4)	Yes (9)
Yoxtheimer et al., 2018 [20]	50	50	21–87	M(30), F(20)	0	8	6	16	Yes (2015)	No	Yes (20)
Viswanathan et al., 2019 [21]	60	57	32–86	M(31), F(29)	0	11	12	19	Yes (2015)	No	Yes (18)

* studies performed in the same study population. Abbreviations: Ref, reference; n, number; M, male; F, female; DIPNECH, diffuse idiopathic neuroendocrine cell hyperplasia; TC, typical carcinoid; AC, atypical carcinoid; HGNECs, high-grade neuroendocrine carcinomas e.g. large cell neuroendocrine carcinoma and small cell lung carcinoma; WHO, world health organization; NE, neuroendocrine; IHC, immunohistochemistry; n/a, not applicable.

**Table 2:** Overview of immunohistochemical features of studies that performed OTP analyses.

Study Year [Ref]	Immunohistochemistry		Outcome (*n* OTP Positive/Total (%))				Staining Scoring	
	Antibody Supplier ^#^	Dilution	DIPNECH	TC	AC	HGNECs	Considered Positive If	Overall Conclusion
Hanley et al., 2018 [18]	Sigma	(1:800)	-	9/9 (100%)	1/6 (17%)	-	Any percentage or intensity of nuclear OTP expression	OTP is a highly sensitive and specific marker for lung carcinoids
Nonaka et al., 2016 * [17]	Atlas	(1:150)	7/7 (100%)	105/123 (85.4%)	10/21 (47.6%)	2/104 (1.9%)	1 + (1–25%), 2 + (25–50%), 3 + (50–75%), 4 + (>75%)	OTP may serve as a useful diagnostic marker for lung carcinoid tumors
Papaxoinis et al., 2018 * [19]	Atlas	(1:150)	16/16 (100%)	117/132 (88.6%)	21/34 (61.8%)	-	More than 5% of the tumor expressed a positive reaction	OTP and TTF1 expression can be used to classify carcinoids into different clusters
Papaxoinis et al., 2017 * [15]	Atlas	(1:150)	-	nOTP < 150 14/69 (20.3%)	nOTP <150 8/17 (47%)	-	H-score (ranging from 0–300)	CD44/nOTP expression is an independent predictor of RFS in patients with radically operated PCs
				cOTP < 150 59/69 (86%)	cOTP < 150 14/17 (82%)	-		
				nOTP > 150 55/69 (80%)	nOTP > 150 9/17 (53%)	-		
				cOTP > 150 10/69 (14.5%)	cOTP > 150 3/17 (18%)	-		
Swarts et al., 2013 [14]	Atlas	(1:800)	-	nOTP 10/225 (4%)	nOTP 3/63 (5%)	nOTP 1/59 (2%)	0 = no staining, 1 = very weak diffuse staining [cytoplasm] or staining in single or very few nuclei, 2 = weak to moderate staining, for nuclear staining in >40% of nuclei, 3–4 = strong to very strong staining in most or all tumor cells, respectively.	OTP and CD44 are powerful prognostic markers for pulmonary carcinoids
				nOTP + cOTP 165/225 (73%)	nOTP + cOTP 28/63 (44%)	nOTP + cOTP 4/59 (7%)		
				cOTP 17/225 (8%)	cOTP 15/63 (24%)	cOTP 8/59 (14%)		
Yoxtheimer et al., 2018 [20]	Sigma	(1:800)	-	4/8 (50%)	1/6 (17%)	1/16 (6.3%)	Min. 5% of tumor cell positivity of 3 + staining intensity	OTP may be used to grade pulmonary NETs and differentiate them from low-grade NETs originating in other sites
Viswanathan et al., 2019 [21]	Sigma	(1:800)	-	9/11 (82%)	8/12 (80%)	0/19 (0%)	Tumor showed >1 + OTP staining in >5% of the tumor within the specimen	OTP is a promising highly sensitive and specific marker for primary pulmonary carcinoid tumors

* studies performed in the same study population. ^#^ All studies used the rabbit anti-OTP polyclonal antibody clone HPA039365. Abbreviations: Ref, reference; n, number; OTP, orthopedia homeobox; DIPNECH, diffuse idiopathic neuroendocrine cell hyperplasia; TC, typical carcinoid; AC, atypical carcinoid; HGNECs, high-grade neuroendocrine carcinomas e.g., large cell neuroendocrine carcinoma and small cell lung carcinoma; TTF1, thyroid transcription factor 1; CD44, cell-surface glycoprotein; nOTP, nuclear OTP expression; cOTP, cytoplasmic OTP expression; RFS, Relapse free survival; PCs, pulmonary carcinoids; NETs, neuroendocrine tumors.
